# Relationship between Femoral Proximal Bone Quality Assessment by MRI IDEAL-IQ Sequence and Body Mass Index in Elderly Men

**DOI:** 10.3390/tomography10050062

**Published:** 2024-05-20

**Authors:** Kashia Goto, Daisuke Watanabe, Norikazu Kawae, Takahiro Nakamura, Kazuki Yanagida, Takahiro Yoshida, Hajime Kajihara, Akio Mizushima

**Affiliations:** 1 Department of Palliative Medicine, Juntendo University Graduate School of Medicine, Tokyo 113-8421, Japan; k.goto.ew@juntendo.ac.jp (K.G.); n.kawae.sh@juntendo.ac.jp (N.K.); akiom@juntendo.ac.jp (A.M.); 2 Department of Urology, Koto Hospital, Tokyo 136-0072, Japan; yanagidak@med.teikyo-u.ac.jp (K.Y.); t.yoshida@med.teikyo-u.ac.jp (T.Y.); 3 Department of Molecular and Cellular Therapeutics, Juntendo University Graduate School of Medicine, Tokyo 113-8421, Japan; 4 Department of Radiology, Koto Hospital, Tokyo 136-0072, Japan; ta20121212@yahoo.co.jp; 5 Department of Orthopedic Surgery, Koto Hospital, Tokyo 136-0072, Japan; h.kajihara.jk@juntendo.ac.jp

**Keywords:** prostate cancer, MRI IDEAL-IQ, androgen deprivation therapy, bone health, bone marrow reconversion, cancer treatment-induced bone loss

## Abstract

Background: Bone assessment using the MRI DEAL-IQ sequence may have the potential to serve as a substitute for evaluating bone strength by quantifying the bone marrow hematopoietic region (R2*) and marrow adiposity (proton density fat fraction: PDFF). Higher body mass index (BMI) is associated with increased bone mineral density (BMD) in the proximal femur; however, the relationship between BMI and R2* or PDFF remains unclear. Herein, we investigated the correlation between BMI and MRI IDEAL-IQ based R2* or PDFF of the proximal femur. Methods: A retrospective single-cohort study was conducted on 217 patients diagnosed with non-metastatic prostate cancer between September 2019 and December 2022 who underwent MRI. The correlation between BMI and R2* or PDFF of the proximal femur was analyzed using Spearman’s rank correlation test. Results: Among 217 patients (median age, 74 years; median BMI, 23.8 kg/m^2^), there was a significant positive correlation between BMI and R2* at the right and left proximal femur (r = 0.2686, *p* < 0.0001; r = 0.2755, *p* < 0.0001, respectively). Furthermore, BMI and PDFF showed a significant negative correlation (r = −0.239, *p* = 0.0004; r = −0.2212, *p* = 0.001, respectively). Conclusion: In elderly men, the increased loading on the proximal femur due to elevated BMI was observed to promote a decrease in bone marrow adiposity in the proximal femur, causing a tendency for a transition from fatty marrow to red marrow with hematopoietic activity. These results indicate that the MRI IDEAL-IQ sequence may be valuable for assessing bone quality deterioration in the proximal femur.

## 1. Introduction

Prostate cancer is the second most common cancer in men worldwide. An estimated 1.1 million men were diagnosed with prostate cancer in 2012 globally, accounting for 15% of all cancers diagnosed in men. As the population worldwide ages, the incidence of prostate cancer is expected to continue to increase [1,2]. Androgen deprivation therapy (ADT) is a key therapy in the treatment of prostate cancer, which is more common in elderly patients and progresses relatively slowly compared to other cancers. However, ADT is known to affect bone metabolism, leading to cancer treatment-induced bone loss (CTIBL), consequently reducing the quality of life in elderly men [3,4].

CTIBL increases the frequency of fractures and shortens the survival period [3]. Urologists assess bone health via bone mineral density (BMD) measurements using dual-energy X-ray absorptiometry (DXA) and bone metabolism marker measurement through blood tests. However, the proportion of patients with prostate cancer who undergo these screenings in practice where ADT has been initiated is insufficient. Many patients who require treatment for decreased bone strength remain untreated, and preventable fractures can occur, becoming a significant issue [5,6].

The bone marrow is divided into the hematopoietic red (hematopoietic marrow) and hematopoietically inactive yellow (fatty marrow) marrows. Shortly after birth, a physiological transition occurs from red to yellow marrow, starting from the distal parts of the limbs and gradually progressing towards the proximal parts with increasing fatty marrow transformation. In adults, red marrow is limited to specific areas such as the vertebral bodies, sternum, ilium, ribs, cranial bones, and proximal femur [7]. Bone loss with fatty myelination is considered a characteristic of the pathophysiology of senile and postmenopausal osteoporosis. Patients with osteoporosis or osteopenia had significantly higher bone marrow fat content than age-matched patients with normal bone density; there was a clear association between bone marrow fat and bone density measured by DXA. Furthermore, the increase in bone marrow fat has been shown to be associated with decreased bone density owing to a preferential increase in saturated fat over unsaturated fat [8]. A study of menopausal women showed that type 2 diabetes and fragility fracture complications cause changes in bone marrow fat composition, resulting in low unsaturated and high saturated fat compositions [9]. Thus, DXA and MRS may be challenging tools for clinical fracture risk assessment, and noninvasive MRI-based analysis of bone marrow fat composition is a novel potential alternative. Recently, the iterative decomposition of water and fat with echo asymmetry and least-squares estimation (IDEAL-IQ) has attracted much attention, as advances in MRI technology have made it possible to quantify iron and fat. The evaluation of iron and fat accumulation in the liver by IDEAL-IQ has been utilized for noninvasive clinical diagnosis of hepatic iron overload and fatty liver [10,11]. IDEAL-IQ is an imaging method that receives echo signals from six different echo times (TE) and calculates T2* and multi-peak fat content from the increased information content. The principle of this method is based on IDEAL (three-point Dixon method), which is a water/fat separation technique. By using the six-point Dixon method, an R2* (reciprocal of T2*) map is created and T2* correction is performed, enabling accurate water/fat separation. The R2* map is considered to be an image that indirectly maps T2* attenuation due to iron deposition. In bone evaluation by IDEAL-IQ, R2*, which indicates iron accumulation, reflects the hematopoietic region (red marrow) in the bone marrow. The image presenting only the fat signal is called the proton density fat fraction (PDFF) and indicates fatty myelination (yellow marrow) [12,13,14]. R2* and PDFF measured by IDEAL-IQ may be used as a non-invasive bone-density-independent fracture risk assessment. Numerous studies have considered the appropriate body mass index (BMI) to maintain BMD, aiming to gather information for lifestyle guidance to preserve the bone health of the elderly. The International Osteoporosis Foundation (IOF) identifies being underweight (BMI < 19 kg/m^2^) as a risk factor for osteoporosis. In cases where frailty coexists, the IOF emphasizes increased fracture risk and recommends engaging in weight-bearing exercises on the hips and muscle strengthening programs about 3–4 times a week for approximately 30 min/session [15]. Generally, a higher BMI is associated with increased BMD in the proximal femur [16]. However, no data on the relationship between BMI and indicators of fracture risk assessment, such as R2* and PDFF, exist. Thus, this study aimed to accumulate additional cases from preliminary research by Watanabe et al. [17], investigate the R2* and PDFF of the proximal femur for the first time, and explore their correlations with BMI.

## 2. Materials and Methods

### 2.1. Statement on Ethics

This study was approved by the Koto Hospital Ethical Review Committee (Approval number 202221; approved on 18 February 2022). This study was conducted following the Declaration of Helsinki proposed in 1964 and its subsequent revisions.

### 2.2. Study Design

Data were extracted from the electronic medical records system database for 238 patients diagnosed with prostate cancer through prostate biopsy at our facility between September 2019 and December 2022. Exclusion criteria included patients with bone metastasis and patients without MRI examination. All 217 patients registered in this study had castration-naive prostate cancer. Age, height, weight, and BMI were retrospectively examined. Within this cohort, no patient had a history of osteoporosis treatment or femoral fractures.

### 2.3. MRI Examination

All MRI examinations were performed using a 1.5T MRI system (SIGNA Voyager 1.5T, Ver.26, GE Healthcare; Tokyo, Japan) with a 16-channel phased-array surface coil. Following the conventional 3-plane localizer, axial and coronal T2-weighted images (repetition time [TR], 2515 ms; echo time [TE], 100 ms; field of view [FOV], 220 mm × 220 mm; slice thickness, 3 mm; matrix, 320 × 224; number of excitations [NEX], 2) and axial and coronal T1-weighted images (TR, 470 ms; TE, 18 ms; FOV, 220 mm × 220 mm; slice thickness, 3 mm; matrix, 320 × 192; NEX, 2) were acquired using a fast spin echo sequence for clinical interpretation. Subsequently, diffusion-weighted imaging was performed axially (TR, 6912 ms; TE, 73.1 ms; FOV, 320 mm × 320 mm; slice thickness, 3 mm; matrix, 96 × 96; NEX, 8; b-value, 1500). Finally, IDEAL-IQ imaging was performed in the coronal direction using scan parameters: TR, 13.1 ms; TE, 5.1 ms; FOV, 400 mm × 400 mm; slice thickness, 8 mm; flip angle, 7°; echo train length, 6; matrix, 160 × 160; and NEX, 1. The scan duration was 34 s.

### 2.4. Evaluation of R2* and PDFF

**Figure 1:**
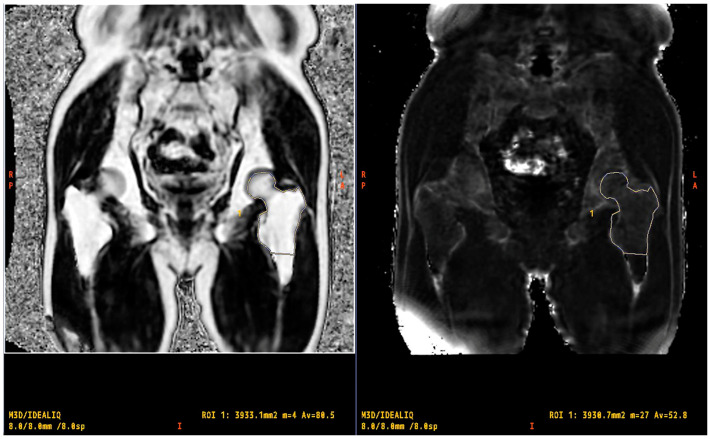
Region of interest including the proximal femur (from the femoral head to the shaft).

The manual trace method was used to trace the proximal femur using a method that passes through a defined area of the proximal femur. IDEAL-IQ imaging was processed using software integrated into the MRI system, generating water, fat, in-phase, out-of-phase, R2*, and fat fraction maps. For the quantification of R2* and PDFF using the IDEAL-IQ sequence at the proximal femur, a free-form region of interest was manually drawn, encompassing the femoral head, neck, greater trochanter, and shaft (up to the distal end of the lesser trochanter), which was analyzed (Figure 1).

### 2.5. Statistical Analysis

For the aggregation of continuous variables, median and interquartile range (IQR) were utilized, while for categorical variables, count and percentages were used. The assessment of correlations between continuous variables involved using Spearman’s rank correlation test, including the analysis of scatter plots and measurement of the Spearman rank correlation coefficient.

Statistical significance was set at *p* < 0.05. All statistical analyses were conducted using JMP^®^ Pro 17 software (SAS Institute Inc., Cary, NC, USA).

## 3. Results

### 3.1. Patient Demographics

Table 1 presents the characteristics of the patients included in this study. The median (IQR) age and BMI for the 217 patients were 74 years (68–80 years) and 23.8 kg/m^2^ (21.9–26 kg/m^2^), respectively. Among the 217 patients, 42 (19.3%) had diabetes, 61 (28.1%) had hypertension, and 54 (24.9%) had dyslipidemia. Additionally, 54 patients (24.9%) were smokers.

### 3.2. Correlation between BMI and IDEAL-IQ PDFF

**Figure 2:**
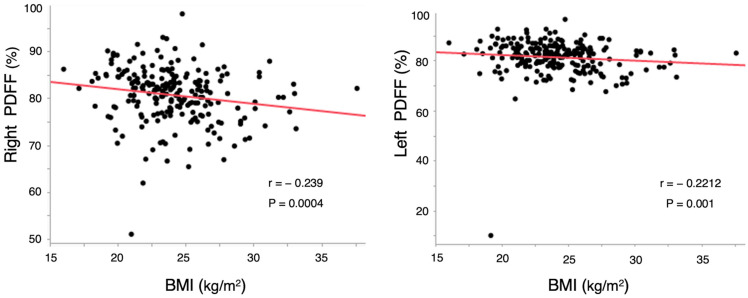
Correlation between BMI and PDFF. BMI, body mass index; PDFF, proton density fat fraction.

The correlation between BMI and PDFF in the proximal femur assessed using IDEAL-IQ was analyzed using Spearman’s rank correlation test. Scatter plots of Spearman rank correlation are shown in Figure 2. The mean PDFF values in the proximal femur were 82.3% and 81.5% for the right and left sides, respectively (Table 1). PDFF in the right proximal femur demonstrated a significant negative correlation with BMI (Figure 2, r = −0.239, *p* = 0.0004). Similarly, PDFF in the left proximal femur showed a significant negative correlation with BMI (Figure 2, r = −0.2212, *p* = 0.001). In a partial correlation analysis considering confounding factors, R2* was also significantly correlated with BMI (Table 2).

### 3.3. Correlation between BMI and IDEAL-IQ R2^*^

**Figure 3:**
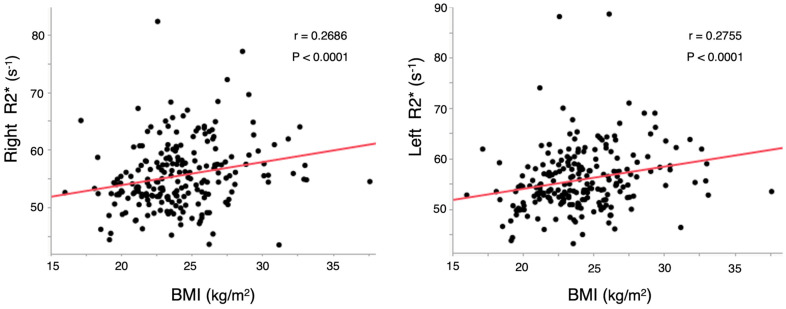
Correlation between BMI and R2*. BMI, body mass index.

The correlation between BMI and R2* in the proximal femur assessed using IDEAL-IQ was analyzed using Spearman’s rank correlation test. Scatter plots of Spearman’s rank correlation are shown in Figure 3. The mean PDFF values in the proximal femur were 54.7 S^−1^ and 54.5 S^−1^ for the right and left sides, respectively (Table 1). R2* in the right proximal femur demonstrated a significant positive correlation with BMI (Figure 3, r = 0.2686, *p* < 0.0001). Similarly, R2* in the left proximal femur showed a significant positive correlation with BMI (Figure 3, r = 0.2755, *p* < 0.0001). However, in the partial correlation analysis considering the confounding factors shown in Table 2, the correlation between PDFF and BMI was no longer significant, but the negative correlation trend remained.

## 4. Discussion

In this study, R2* and PDFF in the proximal femur were measured by using the IDEAL-IQ sequence in Japanese males, and their correlations with BMI were investigated for the first time. The PDFF (bone fat accumulation) in the proximal femur on both sides showed a significant negative correlation with BMI, while the R2* (bone iron accumulation) in the proximal femur on both sides exhibited a significant positive correlation with BMI in Spearman’s correlation analysis. In partial correlation analysis considering confounding factors, R2* was significantly correlated with BMI, but the correlation between PDFF and BMI was no longer significant, but the negative correlation trend remained. A positive correlation exists between BMI and proximal femur BMD [16], with a negative correlation between BMD and marrow fat accumulation [18,19,20]. The positive correlation between BMI and proximal femur BMD may be attributed to the importance of weight-bearing and muscle loading on bone, leading to increased BMD and quality improvement in the proximal femur. The correlation results between BMI and bone quality in the proximal femur evaluated using MRI IDEAL-IQ in this study were similar to the results of studies on correlations between BMI and BMD. Specifically, the result suggests that as BMI increases, bone strength in the proximal femur also increases. Using MRI IDEAL-IQ for bone quality assessment indicates the potential utility of this method as a non-invasive measure of bone strength.

While osteoporosis primarily affects postmenopausal women, it is a significant disease in men as well, associated with major fractures impacting the QOL and mortality rates [21]. Aging in men is related to decreased BMD and osteoporotic fractures [22]. While low BMI in older individuals is a risk factor for fractures, the association between high BMI and fractures is unclear. Nielson et al. found that the correlation between BMI and non-vertebral fracture risk in men ≥ 65 years was non-significant when adjusting for mobility limitations and walking pace. However, obesity is associated with an increased risk of fractures when bone mineral density is maintained at a constant [16]. Gilsanz et al. demonstrated a negative correlation between visceral fat and bone structure and strength, while subcutaneous fat showed a positive correlation. Their findings suggest that subcutaneous fat may benefit bone structure and strength [23].

Amid the growing interest in the relationship between BMD, bone strength, and fat, research on bone marrow fat, reflecting the bone marrow environment, has been advancing. There are many pathophysiological relationships between bone and fat, and osteoblasts and adipocytes are differentiated from the same mesenchymal stem cells. Aging, which is a major cause of osteoporosis, and estrogen deficiency are thought to cause a decrease in the osteogenic potential of mesenchymal stem cells and an increase in their adipogenic potential. Patsch et al. showed that bone marrow fat composition differs in menopausal women with and without fragility fractures and in those with and without type 2 diabetes [9]. Bone marrow fat has tissue characteristics and is metabolically different from peripheral adipose tissue; however, its functional significance remains unknown. An inverse correlation between bone marrow fat tissue and BMD has been reported [24,25]. Bone marrow fat is known to consist mainly of saturated, monounsaturated, and polyunsaturated triglycerides. In a study by Yeung et al., bone marrow fat content increased with decreased bone density, with a relative increase in saturated fat composition over unsaturated fat; further, they showed that adipocytes and osteoblasts share common progenitor cells (mesenchymal stem cells) in the bone marrow and that increased adipogenesis may be associated with decreased osteoblasts [8]. The molecular mechanisms for the interaction between adipogenesis and osteogenesis in bone marrow are not fully understood. Accumulating evidence to date indicates that mature adipocytes may inhibit osteoblast proliferation via the release of polyunsaturated fatty acids. In addition, long-chain fatty acids and oxidized derivatives of fatty acids have been shown to act as ligands for the peroxisome proliferator-activated receptor (PPARγ), which is a nuclear hormone receptor, resulting in the promotion of adipogenesis and the inhibition of osteogenesis in bone marrow stromal cells [26].

Yu et al. investigated the mechanism by which fatty marrow conversion progresses with decreased BMD. They demonstrated that aberrant lineage specification of skeletal stem cells (SSCs), a subset of bone marrow stromal cells that become precursor cells for osteoblasts and adipocytes, is associated with decreased BMD and increased bone marrow fat tissue. They identified peroxisome-proliferator-activated receptor γ coactivator 1-α (PGC-1α) as a determinant of the fate of human and mouse SSCs. Furthermore, they found that PGC-1α expression decreases with aging. PGC-1α deficiency in mouse SSCs promoted bone marrow fat tissue accumulation, impaired bone formation, and indirectly stimulated bone resorption [20]. Conversely, PGC-1α induction suppressed the reduction in bone mass and bone marrow fat tissue accumulation in patients with osteoporosis [20]. Handschin and Almeida demonstrated that PGC-1α deficiency leads to inflammation mediated by NF-κB, resulting in oxidative stress [27,28,29,30]. These findings suggest a potential association between PGC-1α deficiency in osteoporosis and age-related bone disorders and abnormal fate determination of SSCs due to inflammation [20].

In this study, an increase in BMI was associated with a decrease in PDFF and an increase in R2* in Japanese men with an average age of 74 years. Bone marrow is spontaneously replaced from red marrow to fat marrow shortly after birth, but aging causes fat marrow to be reconverted to red marrow; thus, this process is reversible. Małkiewicz et al. have reported on this age-related bone marrow reconversion [31]. They noted that hematopoiesis is stimulated by factors such as smoking (heavy smokers, >2 pack years), high-intensity sports (long-distance running, free diving), obesity and associated respiratory disorders, diabetes, chronic diseases associated with anemia (chronic inflammatory diseases), and treatment with hematopoietic growth factors (such as G-CSF formulations), resulting in the reconversion from yellow adipose marrow to red marrow. Therefore, understanding the distribution of red and yellow adipose marrow, dependent on age, complications, and clinical symptoms, is crucial for interpreting musculoskeletal MRI [31]. Among the patients included in this study, 42 (19.3%) had diabetes, 54 (24.9%) were smokers, and the mean BMI was 23.8 kg/m^2^ (21.9–26.0 kg/m^2^). The increased load on the proximal femur owing to increased BMI may have triggered bone energy metabolism. This increased energy demand could have led to higher oxygen consumption within the bone marrow, resulting in a hypoxic state, and this could have caused bone marrow reconversion from yellow adipose to red marrow. Hypoxia-inducible factor-1α (HIF-1α) is upregulated owing to the stability of PGC-1α in a hypoxic environment [32,33]. Ishii et al. reported that the hypoxic state of bone marrow induced by G-CSF administration in mice results in the release of fibroblast growth factor 23 (FGF-23) from erythroblasts, promoting the mobilization of hematopoietic precursor cells [34]. While FGF-23 secreted from bones is known as a phosphaturic hormone, an increase in FGF-23 decreases the concentration of 1,25(OH)_2_D and increases parathyroid hormone (PTH) levels [35,36]. In the study by Mirza et al., increased serum FGF-23 levels in elderly men were identified as an independent predictor of fracture risk, even after adjustment for age, BMI, BMD, glomerular filtration rate, Vitamin D, and PTH [37]. The relationship between FGF-23 and fracture risk was nonlinear, and FGF-23 levels above 55.7 pg/mL were associated with an increased risk of hip and non-vertebral fractures [37]. However, measuring FGF-23 is not common clinically, and considering FGF-23 was challenging in this study. HIF-1α is a direct transcriptional activator of FGF-23 [38]. The hypoxic conditions in the proximal femur bone marrow caused by high BMI may induce HIF-1α, potentially leading to an increase in fracture risk due to FGF-23 secretion and a decrease in 1,25(OH)_2_D concentration. Inducing PGC-1α via HIF-1α may result in the reconversion of bone marrow fatty tissue to hematopoietic stem cells. In the hypoxic environment of the bone marrow caused by high BMI, HIF-1α and HIF-2α might be induced. HIF-2α promotes endogenous erythropoietin production, iron absorption in the intestine, and iron transport to the bone marrow, stimulating red blood cell production in hematopoietic stem cells in the bone marrow [39,40]. Based on the results of this study, it was hypothesized that these mechanisms are directly or indirectly involved in the positive correlation between BMI and R2* and the reassignment of fat marrow to red marrow. Unfortunately, HIF is a transcriptional inducer and cannot be measured accurately. No studies have yet examined the effect of hypoxic conditions caused by an increased BMI on bone marrow fat composition, and the detailed conditions for bone marrow remodeling are not known. Regarding the effect of BMI on bone quality, Palermo et al. suggest that there seems to be a “U”-shaped relationship between BMI and fracture risk and that obesity can be detrimental to bone health or may play a protective role in bone health [41]. In particular, systemic inflammation due to several pathologies, including aging, insulin resistance, metabolic syndrome, diabetes mellitus, and sex hormone deficiency, appears to compromise the balance of body composition leading to bone loss. Because obesity is a complex disease with a multifactorial etiology and most existing studies are observational, it is possible to suggest a potential correlation between obesity and bone metabolism, but this correlation is difficult to demonstrate [41]. In this study, BMI was significantly correlated with PDFF in Spearman’s correlation analysis, but the correlation was no longer significant in the partial correlation analysis considering the confounding factors, but the negative correlation trend remained. Further research, including non-clinical studies, should explore the relationship between BMI and BMD or quality.

This study has some limitations. First, this was a retrospective study focusing only on Japanese patients, and the number of enrolled Japanese male patients was relatively small. Second, this study was cross-sectional, and post-ADT MRI evaluations were unavailable. Evaluating the relationship between baseline and post-ADT IDEAL-IQ images and BMD could be clinically significant in assessing the progression prediction of cancer treatment-related bone loss. Third, pelvic MRI data were obtained from elderly male patients with a mean age of 74 (68–80) years. Although patients who had not undergone DXA were excluded and patients with a history of osteoporosis treatment or previous femur fracture were not included, it was unable to distinguish between patients with healthy bone loss and osteoporosis using DXA BMD. Finally, owing to the small sample size in this study, the analysis was not conducted after excluding confounding factors that could affect BMD, such as alcohol consumption and physical activity. It is likely that using the same MRI device in the facility helped minimize measurement errors.

## 5. Conclusions

This study demonstrated a significant correlation between R2* and PDFF measured using MRI IDEAL-IQ and BMI in Spearman’s correlation analysis. In partial correlation analysis considering confounding factors, R2* was significantly correlated with BMI, but the correlation between PDFF and BMI was no longer significant, but the negative correlation trend remained. Using IDEAL-IQ for bone evaluation with existing pelvic MRI suggests a clinical and non-invasive bone assessment method for determining bone quality deterioration in the proximal femur.

## Figures and Tables

**Table 1:** Patient demographics.

	All Patients
N (%)	217 (100)
Age (years)	74 (68–80)
Height (m)	1.66 (1.62–1.70)
BMI (kg/m^2^)	23.8 (21.9–26)
Body weight (kg)	65 (59.5–72)
MRI IDEAL-IQ data	
R2* (s^−1^) Right	54.5 (51.9–58.45)
Left	54.7 (51.95–58.8)
PDFF (%) Right	81.5 (77.45–84.5)
Left	82.3 (78.1–85)
Diabetes (n, %)	42 (19.3)
Hypertension (n, %)	61 (28.1)
Dyslipidemia (n, %)	54 (24.9)
Smoker (n, %)	54 (24.9)

The number of patients for each item (percentage of the total) is indicated in the right-hand column. For other continuous variable data, the median (interquartile range, IQR) is provided. BMI, Body mass index; IDEAL-IQ, Iterative decomposition of water and fat with echo asymmetry and least square estimation; MRI, Magnetic resonance imaging.

**Table 2:** Partial correlation analysis.

Variable	Age	BMI	R2* Right	PDFF Right	Diabetes	Dyslipidemia
Age						
corr.		−0.1028	−0.1597	−0.0841	0.1182	0.0335
*p*-value		0.1013	0.0106	0.1807	0.0594	0.5948
BMI						
corr.	−0.1028		0.1529	−0.0968	0.1393	0.0505
*p*-value	0.1013		0.0145	0.1232	0.0261	0.4220
R2* Right						
corr.	−0.1597	0.1529		−0.3808	0.0187	−0.0657
*p*-value	0.0106	0.0145		<0.0001	0.7662	0.2957
PDFF Right						
corr.	−0.0841	−0.0968	−0.3808		0.0336	−0.0692
*p*-value	0.1807	0.1232	<0.0001		0.5938	0.2710
Diabetes						
corr.	0.1182	0.1393	0.0187	0.0336		0.2701
*p*-value	0.0594	0.0261	0.7662	0.5938		<0.0001
Dyslipidemia						
corr.	0.0335	0.0505	−0.0657	−0.0692	0.2701	
*p*-value	0.5948	0.4220	0.2957	0.2710	<0.0001	

BMI, body mass index; PDFF, proton density fat fraction.

## Data Availability

Data are contained within the article.

## References

[1] Ferlay J., Soerjomataram I., Dikshit R., Eser S., Mathers C., Rebelo M., Parkin D.M., Forman D., Bray F. (2015). Cancer Incidence and Mortality Worldwide: Sources, Methods and Major Patterns in GLOBOCAN 2012. Int. J. Cancer.

[2] Pakzad R., Mohammadian-Hafshejani A., Ghoncheh M., Pakzad I., Salehiniya H. (2015). The Incidence and Mortality of Prostate Cancer and Its Relationship with Development in Asia. Prostate Int..

[3] Bliuc D., Nguyen N.D., Milch V.E., Nguyen T.V., Eisman J.A., Center J.R. (2009). Mortality Risk Associated with Low-Trauma Osteoporotic Fracture and Subsequent Fracture in Men and Women. JAMA.

[4] Manolagas S.C., O’Brien C.A., Almeida M. (2013). The Role of Estrogen and Androgen Receptors in Bone Health and Disease. Nat. Rev. Endocrinol..

[5] Alibhai S.M.H., Yun L., Cheung A.M., Paszat L. (2012). Screening for Osteoporosis in Men Receiving Androgen Deprivation Therapy. JAMA.

[6] Tanvetyanon T. (2005). Physician Practices of Bone Density Testing and Drug Prescribing to Prevent or Treat Osteoporosis during Androgen Deprivation Therapy. Cancer.

[7] Tanaka O., Takagi S., Matsuura K., Ichikawa T., Kobayashi Y., Nagai J. (1995). [MR Imaging Findings of the Femoral Marrow in Myelodysplastic Syndrome]. Nihon Igaku Hōshasen Gakkai Zasshi.

[8] Yeung D.K.W., Griffith J.F., Antonio G.E., Lee F.K.H., Woo J., Leung P.C. (2005). Osteoporosis Is Associated with Increased Marrow Fat Content and Decreased Marrow Fat Unsaturation: A Proton MR Spectroscopy Study. J. Magn. Reson. Imaging.

[9] Patsch J.M., Li X., Baum T., Yap S.P., Karampinos D.C., Schwartz A.V., Link T.M. (2013). Bone Marrow Fat Composition as a Novel Imaging Biomarker in Postmenopausal Women with Prevalent Fragility Fractures. J. Bone Miner. Res..

[10] Eskreis-Winkler S., Corrias G., Monti S., Zheng J., Capanu M., Krebs S., Fung M., Reeder S., Mannelli L. (2018). IDEAL-IQ in an Oncologic Population: Meeting the Challenge of Concomitant Liver Fat and Liver Iron. Cancer Imaging.

[11] Imajo K., Kessoku T., Honda Y., Hasegawa S., Tomeno W., Ogawa Y., Motosugi U., Saigusa Y., Yoneda M., Kirikoshi H. (2022). MRI-Based Quantitative R2* Mapping at 3 Tesla Reflects Hepatic Iron Overload and Pathogenesis in Nonalcoholic Fatty Liver Disease Patients. J. Magn. Reson. Imaging.

[12] Ma Q., Cheng X., Hou X., Yang Z., Ma D., Wang Z. (2021). Bone Marrow Fat Measured by a Chemical Shift-Encoded Sequence (IDEAL-IQ) in Patients with and without Metabolic Syndrome. J. Magn. Reson. Imaging.

[13] Zeng Z., Ma X., Guo Y., Ye B., Xu M., Wang W. (2021). Quantifying Bone Marrow Fat Fraction and Iron by MRI for Distinguishing Aplastic Anemia from Myelodysplastic Syndromes. J. Magn. Reson. Imaging.

[14] Hernando D., Hines C.D., Yu H., Reeder S.B. (2012). Addressing Phase Errors in Fat-Water Imaging Using a Mixed Magnitude/Complex Fitting Method. Magn. Reson. Med..

[15] International Osteoporosis Foundation (2023). IOF Osteoporrosis Risk Check. https://riskcheck.osteoporosis.foundation.

[16] Nielson C.M., Marshall L.M., Adams A.L., LeBlanc E.S., Cawthon P.M., Ensrud K., Stefanick M.L., Barrett-Connor E., Orwoll E.S., Osteoporotic Fractures in Men Study Research Group (2011). BMI and Fracture Risk in Older Men: The Osteoporotic Fractures in Men Study (MrOS). J. Bone Miner. Res..

[17] Watanabe D., Kimura T., Yanagida K., Yoshida T., Kawae N., Nakamura T., Kajihara H., Mizushima A. (2022). Feasibility of Assessing Male Osteoporosis Using MRI IDEAL-IQ Sequence of Proximal Femur in Prostate Cancer Patients. Aging Male.

[18] Griffith J.F., Yeung D.K.W., Antonio G.E., Lee F.K.H., Hong A.W.L., Wong S.Y.S., Lau E.M.C., Leung P.C. (2005). Vertebral Bone Mineral Density, Marrow Perfusion, and Fat Content in Healthy Men and Men with Osteoporosis: Dynamic Contrast-Enhanced MR Imaging and MR Spectroscopy. Radiology.

[19] Shan B., Ding H., Lin Q., Zuo X., Lin L., Yu D., Hu C. (2022). Repeatability and Image Quality of IDEAL-IQ in Human Lumbar Vertebrae for Fat and Iron Quantification Across Acquisition Parameters. Comput. Math. Methods Med..

[20] Yu B., Huo L., Liu Y., Deng P., Szymanski J., Li J., Luo X., Hong C., Lin J., Wang C.-Y. (2018). PGC-1α Controls Skeletal Stem Cell Fate and Bone-Fat Balance in Osteoporosis and Skeletal Aging by Inducing TAZ. Cell Stem Cell.

[21] Johnell O., Kanis J.A. (2006). An Estimate of the Worldwide Prevalence and Disability Associated with Osteoporotic Fractures. Osteoporos. Int..

[22] Walsh J.S., Eastell R. (2013). Osteoporosis in Men. Nat. Rev. Endocrinol..

[23] Gilsanz V., Chalfant J., Mo A.O., Lee D.C., Dorey F.J., Mittelman S.D. (2009). Reciprocal Relations of Subcutaneous and Visceral Fat to Bone Structure and Strength. J. Clin. Endocrinol. Metab..

[24] Fazeli P.K., Horowitz M.C., MacDougald O.A., Scheller E.L., Rodeheffer M.S., Rosen C.J., Klibanski A. (2013). Marrow Fat and Bone--New Perspectives. J. Clin. Endocrinol. Metab..

[25] Schwartz A.V. (2015). Marrow Fat and Bone: Review of Clinical Findings. Front. Endocrinol..

[26] Nuttall M.E., Gimble J.M. (2004). Controlling the Balance between Osteoblastogenesis and Adipogenesis and the Consequent Therapeutic Implications. Curr. Opin. Pharmacol..

[27] Almeida M., Han L., Ambrogini E., Weinstein R.S., Manolagas S.C. (2011). Glucocorticoids and Tumor Necrosis Factor α Increase Oxidative Stress and Suppress Wnt Protein Signaling in Osteoblasts. J. Biol. Chem..

[28] Handschin C., Spiegelman B.M. (2008). The Role of Exercise and PGC1alpha in Inflammation and Chronic Disease. Nature.

[29] Kim M.S., Yang Y.-M., Son A., Tian Y.S., Lee S.-I., Kang S.W., Muallem S., Shin D.M. (2010). RANKL-Mediated Reactive Oxygen Species Pathway That Induces Long Lasting Ca2+ Oscillations Essential for Osteoclastogenesis. J. Biol. Chem..

[30] Lee N.K., Choi Y.G., Baik J.Y., Han S.Y., Jeong D.-W., Bae Y.S., Kim N., Lee S.Y. (2005). A Crucial Role for Reactive Oxygen Species in RANKL-Induced Osteoclast Differentiation. Blood.

[31] Małkiewicz A., Dziedzic M. (2012). Bone Marrow Reconversion—Imaging of Physiological Changes in Bone Marrow. Pol. J. Radiol..

[32] Bao X., Zhang J., Huang G., Yan J., Xu C., Dou Z., Sun C., Zhang H. (2021). The Crosstalk between HIFs and Mitochondrial Dysfunctions in Cancer Development. Cell Death Dis..

[33] Yuan P., Yang T., Mu J., Zhao J., Yang Y., Yan Z., Hou Y., Chen C., Xing J., Zhang H. (2020). Circadian Clock Gene NPAS2 Promotes Reprogramming of Glucose Metabolism in Hepatocellular Carcinoma Cells. Cancer Lett..

[34] Ishii S., Suzuki T., Wakahashi K., Asada N., Kawano Y., Kawano H., Sada A., Minagawa K., Nakamura Y., Mizuno S. (2021). FGF-23 from Erythroblasts Promotes Hematopoietic Progenitor Mobilization. Blood.

[35] Bacchetta J., Sea J.L., Chun R.F., Lisse T.S., Wesseling-Perry K., Gales B., Adams J.S., Salusky I.B., Hewison M. (2013). Fibroblast Growth Factor 23 Inhibits Extrarenal Synthesis of 1,25-Dihydroxyvitamin D in Human Monocytes. J. Bone Miner. Res..

[36] Portales-Castillo I., Simic P. (2022). PTH, FGF-23, Klotho and Vitamin D as Regulators of Calcium and Phosphorus: Genetics, Epigenetics and Beyond. Front. Endocrinol..

[37] Mirza M.A., Karlsson M.K., Mellström D., Orwoll E., Ohlsson C., Ljunggren O., Larsson T.E. (2011). Serum Fibroblast Growth Factor-23 (FGF-23) and Fracture Risk in Elderly Men. J. Bone Miner. Res..

[38] Zhang Q., Doucet M., Tomlinson R.E., Han X., Quarles L.D., Collins M.T., Clemens T.L. (2016). The Hypoxia-Inducible Factor-1α Activates Ectopic Production of Fibroblast Growth Factor 23 in Tumor-Induced Osteomalacia. Bone Res..

[39] Gupta N., Wish J.B. (2017). Hypoxia-Inducible Factor Prolyl Hydroxylase Inhibitors: A Potential New Treatment for Anemia in Patients with CKD. Am. J. Kidney Dis..

[40] Haase V.H. (2017). HIF-Prolyl Hydroxylases as Therapeutic Targets in Erythropoiesis and Iron Metabolism. Hemodial. Int..

[41] Palermo A., Tuccinardi D., Defeudis G., Watanabe M., D’Onofrio L., Lauria Pantano A., Napoli N., Pozzilli P., Manfrini S. (2016). BMI and BMD: The Potential Interplay between Obesity and Bone Fragility. Int. J. Environ. Res. Public Health.

